# Discovery of survival factor for primitive chronic myeloid leukemia cells using induced pluripotent stem cells

**DOI:** 10.1016/j.scr.2015.10.015

**Published:** 2015-10-31

**Authors:** Kran Suknuntha, Yuki Ishii, Lihong Tao, Kejin Hu, Brian E. McIntosh, David Yang, Scott Swanson, Ron Stewart, Jean Y.J. Wang, James Thomson, Igor Slukvin

**Affiliations:** a Department of Pathology and Laboratory Medicine, University of Wisconsin, Madison, WI 53792, United States; b Department of Medicine, Moores Cancer Center, School of Medicine, University of California, San Diego, La Jolla, CA 92093-0820, United States; c Wisconsin National Primate Research Center, University of Wisconsin, Madison, WI 53715, United States; d Morgridge Institute for Research, Madison, WI 53707, United States; e Department of Cell and Regenerative Biology, School of Medicine and Public Health, University of Wisconsin, Madison, WI 53707, United States; f Department of Molecular, Cellular & Developmental Biology, University of California, Santa Barbara, CA 93106, United States

## Abstract

A definitive cure for chronic myeloid leukemia (CML) requires identifying novel therapeutic targets to eradicate leukemia stem cells (LSCs). However, the rarity of LSCs within the primitive hematopoietic cell compartment remains a major limiting factor for their study in humans. Here we show that primitive hematopoietic cells with typical LSC features, including adhesion defect, increased long-term survival and proliferation, and innate resistance to tyrosine kinase inhibitor (TKI) imatinib, can be generated *de novo* from reprogrammed primary CML cells. Using CML iPSC-derived primitive leukemia cells, we discovered olfactomedin 4 (OLFM4) as a novel factor that contributes to survival and growth of somatic lin^−^CD34^+^ cells from bone marrow of patients with CML in chronic phase, but not primitive hematopoietic cells from normal bone marrow. Overall, this study shows the feasibility and advantages of using reprogramming technology to develop strategies for targeting primitive leukemia cells.

## 1. Introduction

CML is a myeloproliferative disorder characterized by unregulated growth of predominantly myeloid cells, and their subsequent accumulation in the bone marrow and peripheral blood. CML originates in hematopoietic stem cells (HSCs) with t(9;22)(q34;q11.2) translocation, which causes the constitutive expression of the BCR-ABL kinase driving the expansion of leukemic progeny (Holtz et al., 2002; Holyoake et al., 2001; Ramaraj et al., 2004). *Ex vivo* cultures of CML-derived cell lines and primary CML cells, ectopic expression of BCR-ABL in CD34^+^ cells and mouse models have provided important insights into CML pathogenesis and led to the development of targeted therapy for this neoplastic disease with BCR-ABL tyrosine kinase inhibitor (TKI), imatinib (Druker et al., 2006; Druker et al., 2001). Despite these achievements, eradication of CML remains challenging. Although the majority of patients treated with imatinib achieve a complete cytogenetic response, discontinuation of imatinib treatment is commonly associated with relapse (Mahon et al., 2010). Multiple lines of evidence suggest that the major cause of disease persistence is innate resistance of leukemia stem cells (LSCs) to TKIs (Corbin et al., 2011; Graham et al., 2002; Holyoake et al., 2001). Thus, studies of primitive leukemia cells are essential for better understanding leukemia pathogenesis and developing curative therapies for CML. Due to the limited number of BCR-ABL^+^ cells within the most primitive hematopoietic cell compartments (Holyoake et al., 1999; Holyoake et al., 2001; Vargaftig et al., 2012), establishing technologies for *de novo* generation of LSC-like cells would provide a significant benefit to the CML field.

Reprogramming human somatic cells to pluripotency allows for the generation of induced pluripotent stem cells (iPSCs) that behave similarly to embryonic stem cells (ESCs), i.e., they are capable of self-renewal, large-scale expansion, and differentiation toward derivatives of all three germ layers, including blood (Choi et al., 2009b; Park et al., 2008; Takahashi et al., 2007; Yu et al., 2009). Because iPSCs capture the entire genome of diseased cells, they are already being used in modeling human genetic diseases (Grskovic et al., 2011). Recently, we and other groups successfully generated iPSCs from primary CML cells and showed that CML-iPSCs capture the genetic alterations present in leukemia cells, and possess the ability to produce differentiated leukemia cells (Bedel et al., 2013; Hu et al., 2011; Kumano et al., 2012). Here, we tested the hypothesis that reprogramming CML cells to pluripotency and then differentiating them back into blood cells can be used as a novel approach to produce an unlimited number of primitive hematopoietic cells with LSC properties and identify novel primitive leukemia cell survival factors and drug targets. We validated this hypothesis by demonstrating the successful application of the iPSC-based platform to discover OLFM4 as a novel primitive leukemia cell survival factor in patients in the chronic phase of CML. This finding provides a basis for development of novel approaches for treating CML by targeting OLFM4 or OLFM4-mediated signaling pathways in primitive leukemia cells.

## 2. Results

### 2.1. Generation of LSC-like cells from CML-iPSCs

Recently we generated transgene-free iPSCs from the bone marrow mononuclear cells of a patient with a newly diagnosed CML in the chronic phase (CML15 iPSCs and CML17 iPSCs) and showed that these iPSCs capture the entire genome of neoplastic cells, including the unique 4-way translocation between chromosomes 1, 9, 22, and 11 that was present in the patient bone marrow (BM) (Hu et al., 2011). Sequencing analysis revealed that the BCR-ABL translocation in these CML-iPSCs expresses the p210 oncoprotein with a typical b3a2 rearrangement and lack of mutations in the kinase domain (Supplementary Fig. S1a and b). These findings were consistent with the observed sensitivity of parental bone marrow progenitors to imatinib (Supplementary Fig. S1c).

CML LSCs have been identified within the most primitive hematopoietic compartments as cells with long-term culture initiating cell (LTC-IC) or in vivo repopulating activities. (Corbin et al., 2011; Li et al., 2012; Petzer et al., 1996; Sloma et al., 2010; Udomsakdi et al., 1992b) Similar to normal HSCs, CML LSCs express typical markers of primitive hematopoietic cells including, CD34 and CD90, and are negative for hematopoietic lineage markers (lin^−^) and CD45RA (Udomsakdi et al., 1992b). They also have high aldehyde dehydrogenase (ALDH) activity and are able to efflux Rhodamine-123 (Udomsakdi et al., 1992a). Since we previously found that cells with such features can be generated from karyotypically normal iPSCs by coculture with OP9 (Choi et al., 2009a; Choi et al., 2009b; Vodyanik et al., 2005), we used this system to induce hematopoietic differentiation from CML iPSCs.

**Fig. 1:**
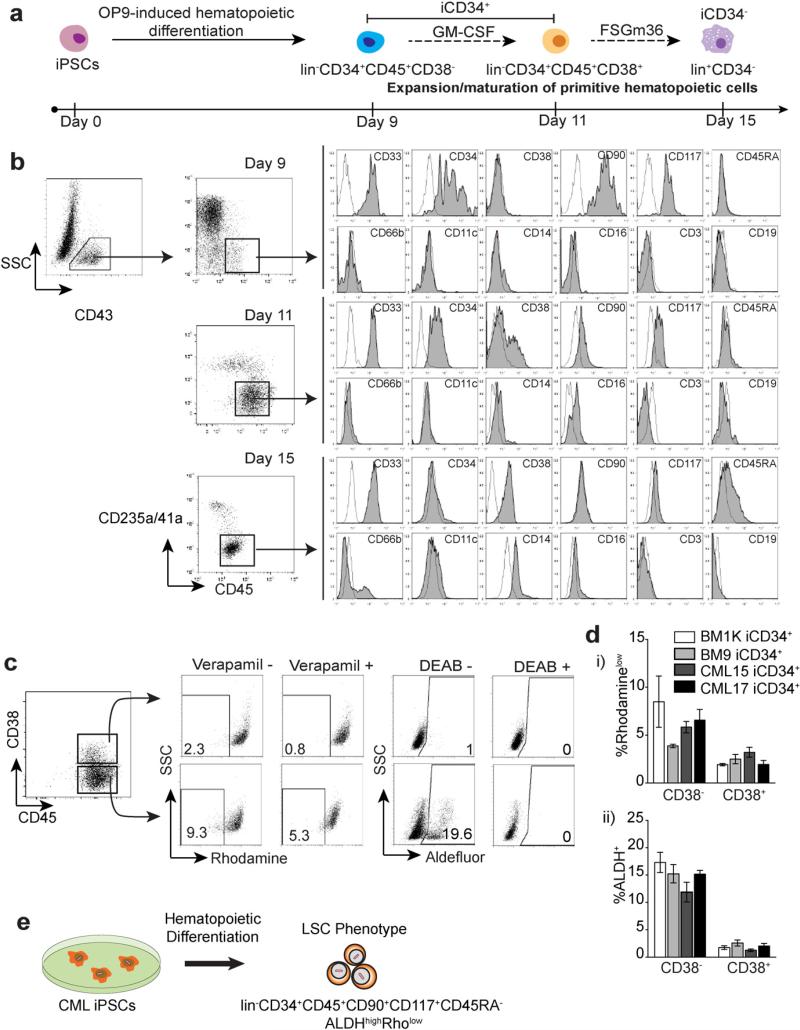
Generation of lin^−^CD34^+^CD43^+^CD45^+^ primitive hematopoietic cells from CML-iPSCs. (a) Schematic diagram of hematopoietic differentiation from iPSCs. Phenotype and its designation are shown under and on the top of cells, respectively. Prefix i indicates iPSC-derived. FSGm36 = Flt3L, SCF, GM-CSF, IL3, IL6. (b) Phenotypes of CD45^+^ cells obtained from CML-iPSCs after differentiation on OP9 (day 9) and following their expansion/differentiation with cytokines in stroma-free cultures (as indicated in A). Plots show isotype control (open) and specific antibody (shaded) histograms. Representative results from three to four independent experiments are shown. (c) and (d) Rhodamine (Rho) efflux and ALDH activity in isolated CML iCD34^+^ cells. Representative dot plots show Rho efflux and ALDH activity in CD34^+^CD38^+^ and CD34^+^CD38^−^ populations (c). Graph shows quantification of Rho efflux (di) and ALDH assay (dii). The values are mean ± SEM of % of Rho^low^ cells (% of Rho^low^_verapamil−_ – Rho^low^_verapamil+_) and ALDH^+^ cells (% of ALDH^+^_DEAB−_ – ALDH^+^_DEAB+_) from three experiments respectively. DEAB = diethylaminobenzaldehyde, ALDH = aldehyde dehydrogenase. (e) Summary of findings.

In OP9 coculture, CML iPSCs and control bone marrow-derived iPSCs (BM1K and BM9) formed CD34^+^CD43^+^ hematopoietic progenitors, including CD235a^−^CD41a^−^CD45^+^ cells that are highly enriched in myeloid progenitors (Hu et al., 2011; Vodyanik et al., 2006) (Fig. 1a and b). As shown in Fig. 1b, CD235a^−^CD41a^−^CD45^+^ cells obtained at day 9 of CML iPSC differentiation had the phenotypic features of CML LSCs including the expression of the primitive hematopoietic cell markers CD34, CD90 and CD117, and an absence of CD38 and CD45RA and line-age markers (lin^−^). Similar to somatic CML LSCs, day 9 CML iPSC-derived CD45^+^ cells had ALDH activity and were able to efflux Rhodamine-123 (Fig. 1c-d). After expansion for 2 days, CD45^+^ cells retained expression of CD34 and CD90 and CD117 stem cell markers and remained lin^−^ and CD45RA^−^. However, up to 25% of the population acquired CD38 expression (Fig. 1b, day 11) and the levels of ALDH^high^ and Rho^low^ cells within CD45^+^ population decreased (Fig. 1c and d). CML iPSC-derived lin^−^CD34^+^CD90^+^CD117^+^CD45^+^38^+/−^ (hereafter referred to as induced CD34^+^; iCD34^+^) cells acquired expression of myeloid lineage-specific markers and lost CD34 expression (Fig. 1b, day 15), i.e. became lin^+^CD34^−^ (hereafter referred to as induced CD34^−^ cells; iCD34^−^) following further culture with hematopoietic cytokines. Thus, we concluded that cells with the LSC phenotype could be generated *de novo* from reprogrammed CML cells (Fig. 1e).

**Fig. 2:**
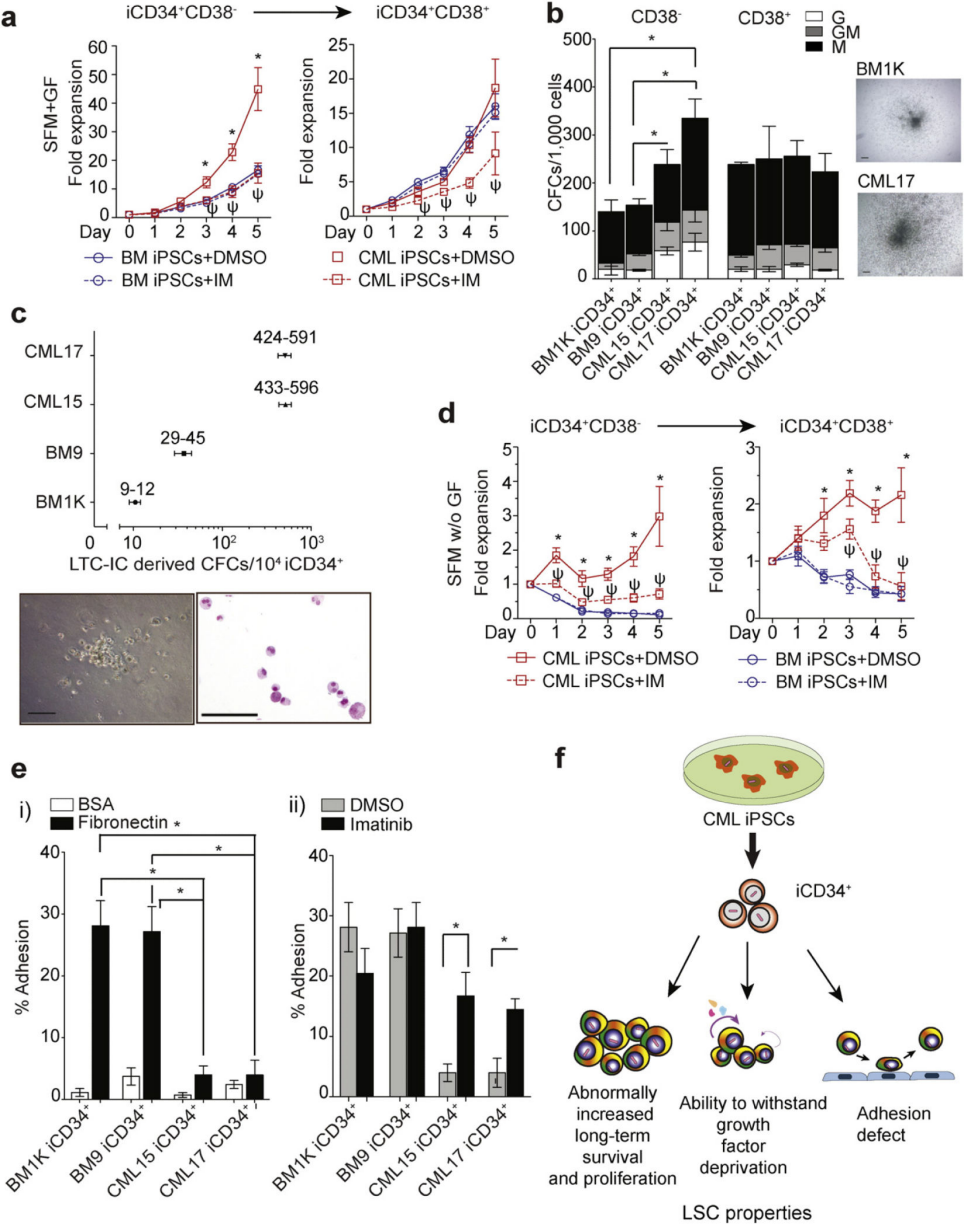
Stem/progenitor cell properties of CML and control BM iPSC-derived iCD34^+^ cells. (a) Expansion of CML and BM iCD34^+^ cells in serum-free medium (SFM) with growth factors (GF) in presence of DMSO (control) or 5 μM imatinib. Results are mean ± SEM from six independent experiments (three from each BM1K and BM9 iCD34^+^, and three from each CML15 and CML17 iCD34^+^). * indicates significant differences between cell counts in BM and CML iCD34^+^ cultures without imatinib (p < 0.05). Ψ indicates significant differences between imatinib treated and untreated CML iCD34^+^ cells (p < 0.05). (b) Colony formation from BM and CML iCD34^+^ cells. Graph shows mean ± SEM from 3 independent experiments. * p < 0.01. Right panels show typical GM colony from BM and CML iCD34^+^ cells. Both, CML iCD34^+^CD38^−^ and iCD34^+^CD38^+^ cells grew much larger colonies in CFC medium as compare to control BM iCD34^+^ cells. (c) LTC-IC assay of CML and BM iCD34^+^ cells. Numbers next to the plot show the total number of CFCs detected in LTC-IC cultures of iCD34^+^ cells. Results are mean ± SEM of two biological replicates performed in triplicates. Representative colony and Wright stained cytospin from the colony formed by CML iCD34^+^ cells after 5 weeks of LTC-IC culture are shown below the chart. Scale bar = 100 μm. (d) Expansion of CML and BM iCD34^+^ cells in serum-free medium (SFM) without growth factors (GF) in presence of DMSO (control) or 5 μM imatinib. Results are mean ± SEM from six independent experiments (three from each BM1K and BM9 iCD34^+^, and three from each CML15 and CML17 iCD34^+^). * indicates significant differences between cell counts in BM and CML iCD34^+^ cultures without imatinib (p < 0.05). Ψ indicates significant differences between imatinib treated and untreated CML iCD34^+^ cells (p < 0.05). (e) Adhesion of BM and CML iCD34^+^ cells to fibronectin or control bovine serum albumin (BSA)-adsorbed plates (ei) and the effect of imatinib on adhesion of iCD34^+^ cells to fibronectin (eii). * p ≤ 0.02. Results are mean ± SEM from three independent experiments. (f) Summary of findings.

Although CML LSCs share many properties with HSCs, they have three distinct characteristics: the increased proliferation and long-term survival (Corbin et al., 2011; Holyoake et al., 1999); the ability to grow in vitro without added cytokines (Jiang et al., 1999); and an adhesion defect (Bhatia et al., 2001; Verfaillie et al., 1992). Therefore, next we evaluated whether *de novo* generated CML iCD34^+^ cells possess similar properties. By analyzing the growth of iCD34^+^ cells in serum-free medium with growth factors, we found an enhanced expansion of iCD34^+^CD38^−^ cells from CML iPSCs relative to similar cells from normal BM iPSCs. Treatment with imatinib decreased expansion of CML iCD34^+^CD38^−^ cells to the level observed in normal BM iCD34^+^CD38^−^ cultures (Fig. 2a). CML iCD34^+^CD38^−^ cells also generated significantly more and larger myeloid colonies as compared to normal BM iCD34^+^CD38^−^ cells (Fig. 2b). Although we did not see differences in the number of colonies between CML and BM cells within CD38^−^ compartment, CML iCD34^+^CD38^−^ cells consistently generated larger colonies. Using the LTC-IC assay, we found that CML iCD34^+^ cells produced a much higher number of LTC-IC-derived CFCs than BM iCD34^+^ cells, indicating an increase in long-term survival (Fig. 2c). In serum-free cultures, CML iCD34^+^ cells were able to withstand growth factor deprivation in both CD38^+^ and CD38^−^ compartments, which was reversed by treatment with imatinib (Fig. 2d). CML iCD34^+^ cells also showed reduced adhesion to fibronectin (Fig. 2ei), which was partially restored by imatinib treatment (Fig. 2eii). Overall, these findings provide strong evidence that iCD34^+^ cells derived from CML iPSCs behave similarly to their somatic LSC counterparts (Fig. 2f).

### 2.2. Induced LSC-like cells are resistant to imatinib

**Fig. 3:**
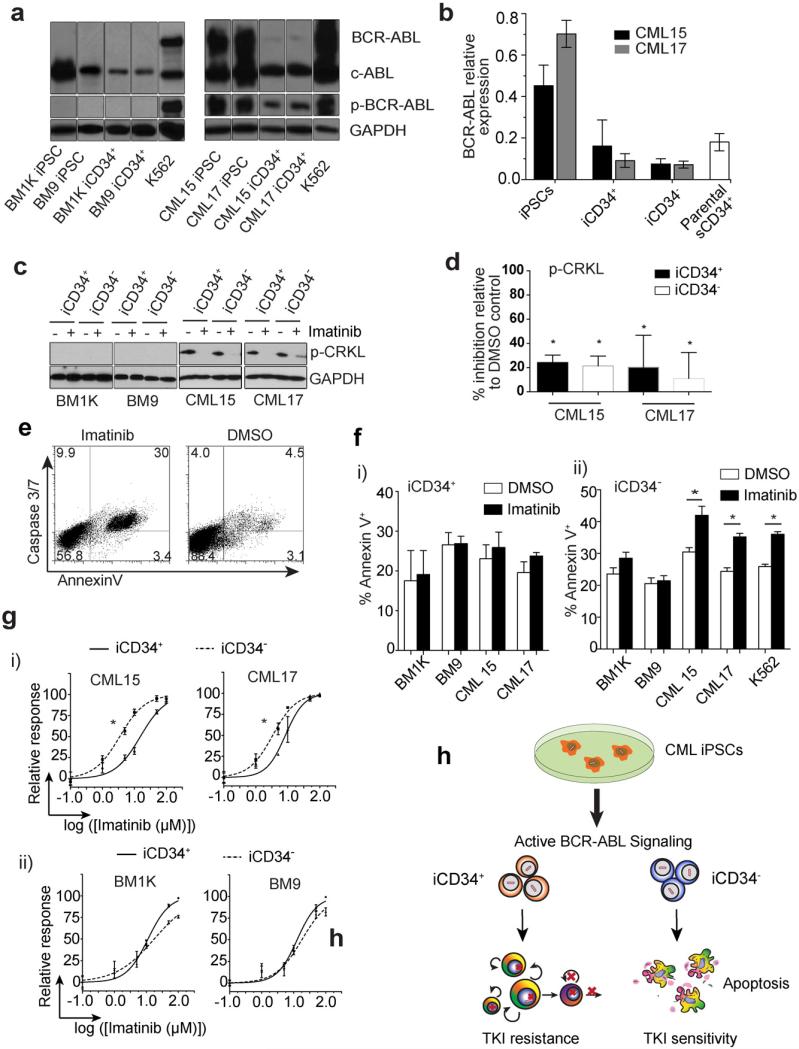
Effects of imatinib on CML iPSC-derived iCD34^+^ cells. (a) Western blot shows expression of BCR-ABL, c-ABL and p-BCR-ABL protein in normal BM iPSCs and CML iPSCs and their hematopoietic derivatives. GAPDH and K562 were used as a loading control and a positive control respectively. (b) qPCR quantification of BCR-ABL mRNA expression in CML iPSCs, their derivatives and paternal sCD34^+^ bone marrow cells. The expression levels were calculated relatively to K562 as calibrator. Results are mean ± SEM from three experiments in duplicate. (c) Western blot shows phospho-CRKL (p-CRKL) expression in iCD34^+^ and iCD34^−^ cells following 4 h treatment with 5 μM imatinib or DMSO. GAPDH was used as a loading control. (d) Quantitative analysis of p-CRKL expression by densitometry. Results are calculated as p-CRKL/GAPDH ratio. Graph shows % of inhibition relative to DMSO control. Results are the mean ± SEM from three independent experiments. (e) Dot plot shows staining of CML iCD34^−^ cells with Annexin V and caspase 3/7 detection agent after treatment with imatinib or DMSO. (f) Apoptosis in BM and CML iCD34^+^ (fi) and iCD34^−^ (fii) cells treated and non-treated with 5 μM imatinib for 24 h. Apoptosis was evaluated by Annexin V staining. Results are the mean ± SEM from three independent experiments. K562 was used as an IM-sensitive control. * p < 0.05. (g) 50% Inhibition concentration (IC_50_) assay from CML (gi) and bone marrow (gii) iCD34^+^ and iCD34^−^ cells is shown as relative response (% of cell death relative to cell death in the upper plateau) versus log concentration of imatinib. * indicates a significant IC_50_ shift (p < 0.05). Results are the mean ± SEM from three independent experiments performed in triplicate. (h) Summary of findings.

The dependence of CML cells on BCR-ABL signaling enables the suppression of the disease by TKIs. However, resistances of CML LSCs to imatinib preclude a complete cure for CML (Corbin et al., 2011; Graham et al., 2002; Holyoake et al., 2001). Analysis of BCR-ABL expression by qPCR and Western blot demonstrated that BCR-ABL mRNA and protein, including phosphorylated protein (p-BCR-ABL) were present in undifferentiated CML iPSCs and their iCD34^+^ and iCD34^−^ progeny (Fig. 3a and b). Interestingly, expression of BCR-ABL mRNA was greater in undifferentiated CML iPSCs when compared to parental bone marrow CD34^+^ cells. However, in iCD34^+^ cells, the level of BCR-ABL expression downregulated to the level observed in parental CD34^+^ cells. Evaluation of the phosphorylation status of the BCR-ABL-specific substrate CRK-like protein (CRKL) revealed the presence of a phosphorylated form of CRKL (p-CRKL) in CML, but not in the control BM iCD34^+^ and iCD34^−^ cells (Fig. 3c), thus providing evidence that BCR-ABL is active in CML iPSC-derived hematopoietic progeny.

Studies in CML patients have shown that imatinib inhibits BCR-ABL kinase and cell proliferation in primitive hematopoietic compartments without affecting survival of LSCs (Copland et al., 2006; Corbin et al., 2011; Graham et al., 2002; Holtz et al., 2002; Schemionek et al., 2010). To find out whether *de novo* generated CML iCD34^+^ cells respond to imatinib in a similar fashion, we evaluated the effect of imatinib on these cells in vitro. After treatment with imatinib for 4 h, p-CRKL dramatically decreased in both primitive iCD34^+^ and in more mature iCD34^−^ blood cells (Fig. 3c and d). This indicates that imatinib efficiently inhibits the majority of BCR-ABL kinase activity in *de novo* generated CML cells, independent of the stage of maturation. The inhibition of BCR-ABL kinase in iCD34^−^ cells by imatinib treatment was associated with increased apoptosis as determined by using Annexin V staining and caspase 3/7 fluorogenic substrate (Fig. 3e and fii). In contrast, imatinib failed to induce apoptosis in CML iCD34^+^ cells (Fig. 3fi), despite the abrogation of p-CRKL signaling in these cells (Fig. 3c and d). The increased resistance to imatinib within the more primitive hematopoietic compartment was also evident from a significant increase of inhibitory concentration 50% (IC_50_) for CML iCD34^+^ cells as compared to CML iCD34^−^ cells (Fig. 3gi). Both, iCD34^+^ and iCD34^−^ cells, generated from normal BM iPSCs, were not affected by imatinib treatment (Fig. 3fi, fii and gii).

To confirm the maturation stage-dependent sensitivity to imatinib, we analyzed the distribution of apoptotic cells within different compartments and generations following expansion of CFSE-labeled CML iCD34^+^ cells. Imatinib treatment of CML iCD34^+^ cell cultures was associated with a significant increase of slowly dividing CD34^+^ cells (generations 2–4) in bulk cultures (Supplementary Fig. S2a and b) and retention of CD34^+^ expression by rapidly dividing cells (generations 5–7; Supplementary Fig. S2c) as compared to non-treated cells. Annexin V staining of CFSE-labeled cell cultures revealed that the most primitive iCD34^bright^ cells were resistant to imatinib-induced apoptosis, while a substantial increase in apoptosis was observed in iCD34^−^ cells and in more mature and proliferative iCD34^dim^ cells the majority of which were CD38^+^ (Supplementary Fig. S2d–f). These findings imply that differentiated CML iCD34^−^ cells became sensitive to imatinib and could not survive in culture, while the most primitive CML iCD34^+^CD38^−^ cells did not undergo apoptosis upon imatinib treatment and were selected to dominate in the culture.

Taken together, these results indicate that CML iCD34^+^ cells reproduce many aspects of drug resistance observed in somatic primitive hematopoietic cells from CML patients in the chronic phase (Fig. 3h).

### 2.3. Identication of olfactomedin 4 as a novel survival factor in LSC-like cells

**Fig. 4:**
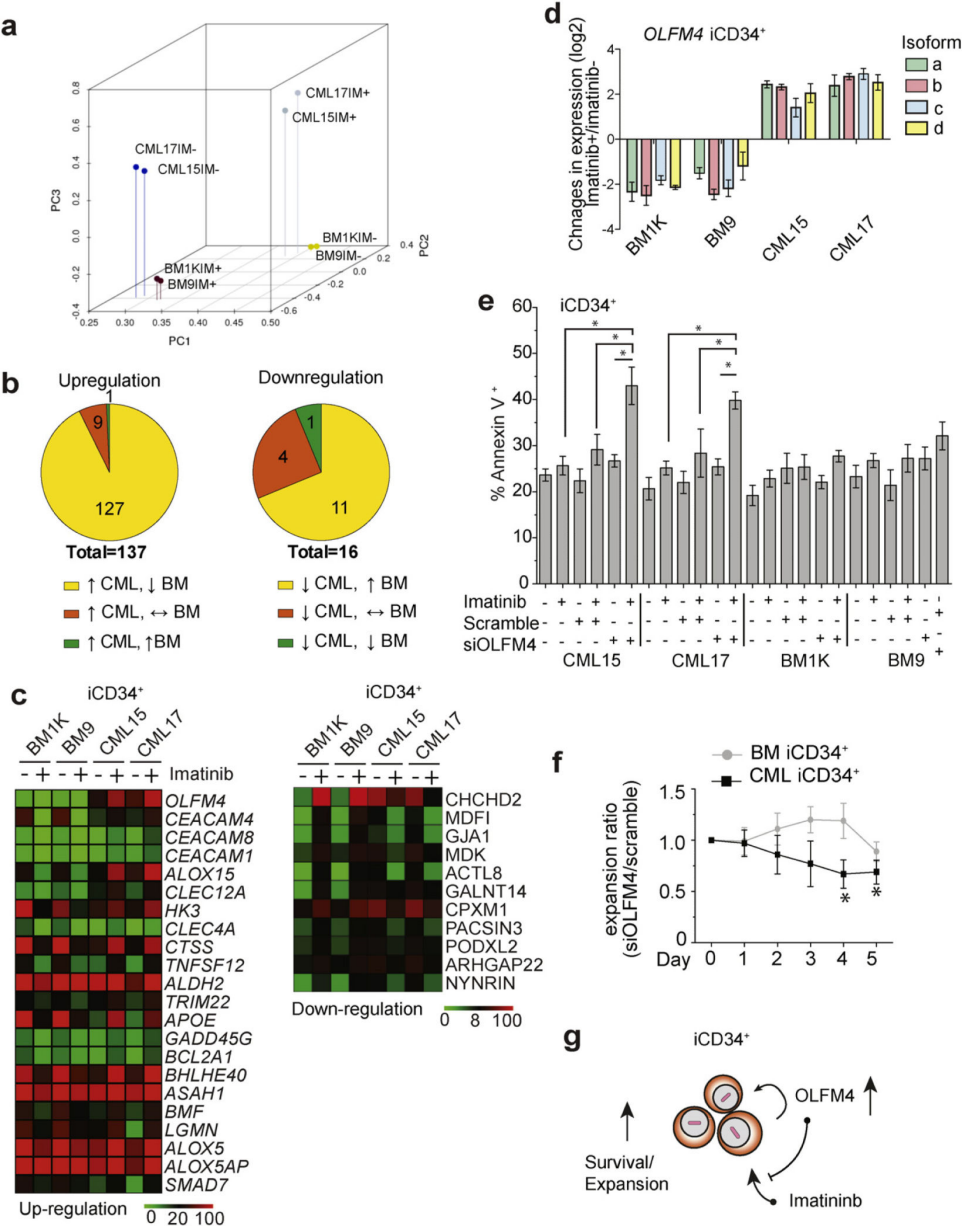
Gene expression analysis reveals set of imatinib-induced genes in CML iPSC-derived iCD34^+^ cells. (a) Principal component (PC) analysis of global gene expression in iCD34^+^ cells treated (IM+) and non-treated (IM−) with imatinib. After treatment with imatinib the position of CML iCD34^+^ cells shifted toward normal non-treated BM iCD34^+^ cells. (b) Selection of candidate genes associated with imatinib resistance. Upper left Venn diagram shows genes significantly upregulated by imatinib in CML iCD34^+^(≥2 fold induction). Upper right Venn diagram shows genes that were significantly suppressed by imatinib (≥2 fold reduction). Genes with tpm < 10 across all studied samples were excluded from analysis. (c) Heat maps showing expression of genes selectively induced or suppressed by imatininb in CML iCD34^+^. The top differentially expressed genes are arranged based on ratio of expression in imatinib-treated CML/imatinib-treated BM iCD34^+^ cells. (d) qPCR analysis of the effect of imatinib treatment on expression of OLFM4 mRNA isoforms in CML and BM iCD34^+^ cells. The results are mean ± SEM of three experiments in duplicate. The expression levels were calculated relative to untreated control. (e) OLFM4 knockdown with siRNA induced apoptosis in imatinib treated CML iCD34^+^ cells. Results are mean ± SEM of three independent experiments. The statistical differences were only observed between CML iCD34^+^ samples treated with imatinib alone and imatinib plus siOLFM4; * p < 0.05 (f) Effect of *OLFM4* knockdown on BM or CML iCD34^+^ cell proliferation in CSSM medium supplemented with growth factors. Graph displays the ratio of cell numbers in cultures treated with siOLFM4 relative to cell numbers in corresponding cultures treated with scrambled siRNA. Results are mean ± SEM of three independent experiments. * indicates significant (p < 0.05) decrease in the expansion cell ratios (siOLFM/scramble siRNA cultures) in CML iCD34^+^ cultures when compared to normal BM controls. (g) Summary of findings.

To find out whether CML iPSC-derived primitive hematopoietic cells can be used to discover novel LSC survival factors and drug targets, we compared the molecular profile of CML and normal BM iCD34^+^ cells that were either treated or not treated with imatinib for 16 h in expansion cultures with cytokines. Similar to the findings with somatic CML CD34^+^ cells (Leng et al. 2013; Li and Dewey 2011), untreated CML iCD34^+^ cells showed significant differences compared to normal BM iCD34^+^ cells in the expression of genes regulating chemotaxis, adhesion, cytokine production, proliferation, programmed cell death, regulation of phosphorylation, and fatty acid metabolism (Supplementary Fig. S3a). These differences included the upregulation of genes associated with cancer development, such as *BCL2*, *CDK6*, *PRKCQ*, *MYCN*, *CDKNA2A*, *GFI1B*, *TP53RK*, and *RASGRP3*, and the downregulation of adhesion molecules *ICAM1*, *ICAM3*, *ITGB2* and *ITGB7* (Supplementary Fig. S3b and Supplementary Table S1). We next evaluated the effect of imatinib on the transcriptome of iCD34^+^ cells by performing principal component analysis (PCA) to define the change in transcriptional distance following drug treatment. As shown in Fig. 4a, after treatment with 5 μM imatinib, CML iCD34^+^ cells moved away from non-treated CML iCD34^+^ cells toward non-treated control BM iCD34^+^ cells in the dimension defined by principal component PC1 and PC2, i.e. became more similar to control BM iCD34^+^ cells, thereby suggesting the important role of BCR-ABL signaling in establishing the unique transcriptional signature of neoplastic cells in CML.

To find candidate genes associated with imatinib resistance, we initially selected genes that were significantly upregulated or downregulated by imatinib in CML iCD34^+^ cells. From the list of upregulated genes, we chose 137 that were induced ≥2 fold by imatinib treatment (Fig. 4b). Then, we selected genes that were upregulated in CML iCD34^+^ cells, but suppressed in BM iCD34^+^ cells, i.e. excluded genes that were upregulated or not affected by imatinib in BM iCD34^+^ cells (Fig. 4b and c and Supplementary Table S2). A similar algorithm was applied for down-regulated genes. From the list of significantly dowregulated genes, we chose those that were suppressed ≥2 fold by imatinib in CML iCD34^+^ (16 genes). We then selected genes that were suppressed in CML iCD34^+^ cells, but upregulated in BM iCD34^+^ cells (Fig. 4b and c and Supplementary Table S2). Overall, based on this selection algorithm we identified 127 upregulated and 11 downregulated genes following imatinib treatment. Upregulation of selected genes (*BCL2A1*, *ALOX15* and *SMAD7*) by imatinib in CML iCD34^+^ cells was confirmed by qPCR analysis (Supplementary Figure S3c). Interestingly, the top-ranking up-regulated gene identified by our algorithm was *OLFM4* (olfactomedin 4), which has been reported to have anti-apoptotic activity in malignancy (Liu et al., 2012; Oh et al., 2011; Zhang et al., 2004). According to AceView analysis (Thierry-Mieg and Thierry-Mieg, 2006), transcription of human *OLFM4* produces 5 different mRNAs (OLFM4a–OLFM4e) encoding four alternatively spliced variants and one unspliced variant. These mRNAs encode four complete proteins, including OLFM4a and OLFM4d secreted isoforms and OLFM4b and OLFM4c cytoplasmic isoforms. OLFM4e isoform is reconstructed from partial mRNA and is not fully characterized (http://www.ncbi.nlm.nih.gov/IEB/Research/Acembly/av.cgi?db=human&c=Gene&l=OLFM4). Using isoform specific primers, we demonstrated the presence of four out of five OLFM4 mRNA isoforms (OLFM4a–OLFM4d) in CML iCD34^+^ cells and the entire set of five mRNA isoforms (OLFM4a–OLFM4e) in CML iCD34^−^ cells (Supplementary Fig. S4a). Consistent with RNAseq data, qPCR analysis revealed the augmentation of OLFM4 expression by imatinib in CML iCD34^+^ cells, including all four OLFM4a–OLFM4d mRNA isoforms (Fig. 4d).

To evaluate the potential role of OLFM4 in survival of CML iCD34^+^ cells, we tested the effect of OLFM4 knocking-down on iCD34^+^ cell apoptosis using *OLFM4*-targeted small interference RNA (siOLFM4) composed of four *OLFM4*-specific siRNAs. Transfection of CML iPSC-derived hematopoietic cells with this set of siRNAs enables 60%–85% reduction in expression of all five OLFM4 mRNA isoforms (Supplementary Fig. S4b). As shown in Fig. 4e, imatinib or siOLFM4 had no effect on CML iCD34^+^ cell apoptosis. However, siOLFM4 treatment in the presence of imatinib induced significant increase of apoptosis in CML, but not normal bone marrow iCD34^+^ cells. In addition, OLFM4 knockdown selectively inhibited expansion of CML iCD34^+^ cells in vitro (Fig. 4f). These findings imply that OLFM4 is involved in regulation of imatinib resistance and survival of *de novo* generated primitive CML cells (Fig. 4g).

### 2.4. OLFM4 is essential for somatic primitive leukemia cell survival in vitro and in vivo engraftment

**Fig. 5:**
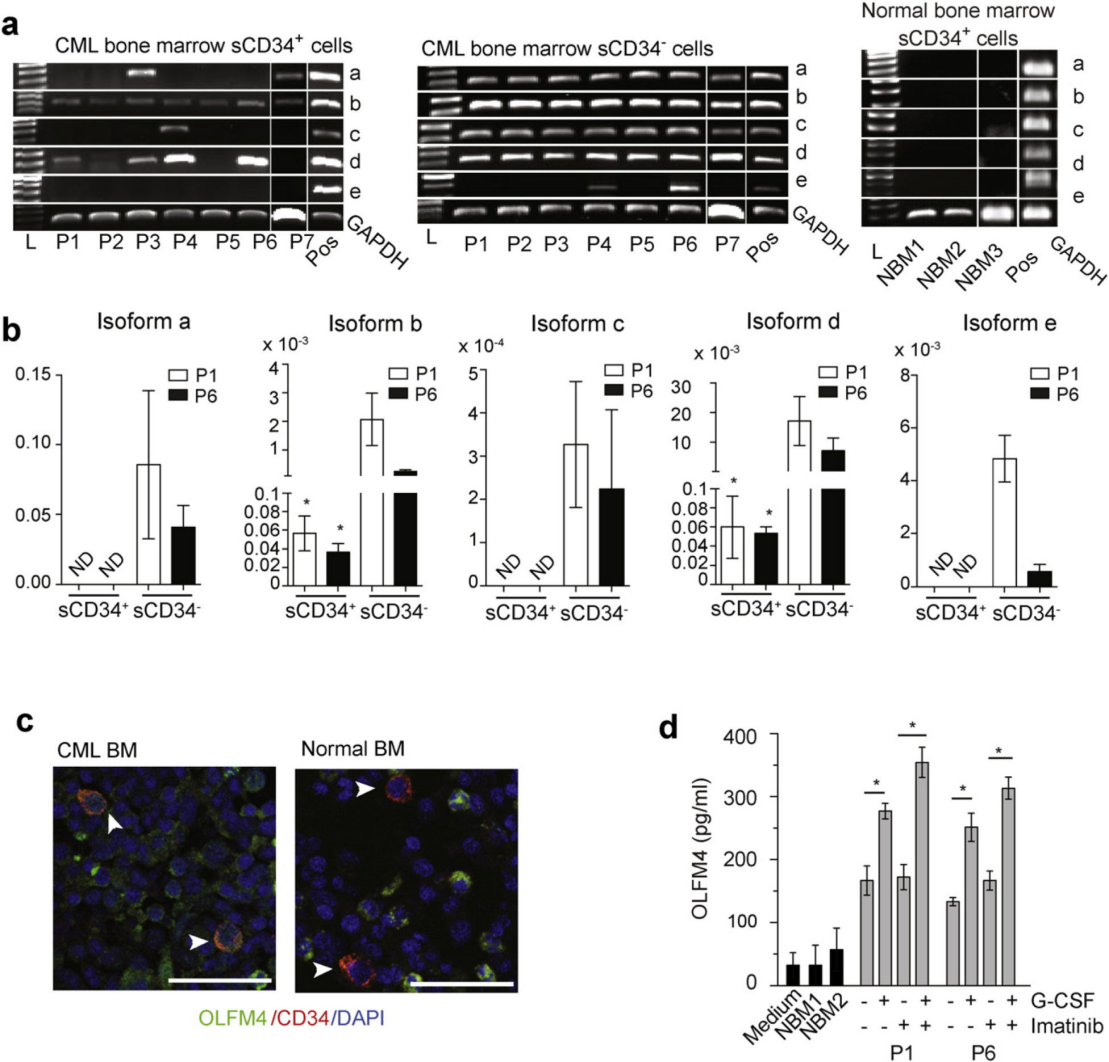
Expression of OLFM4 in somatic CD34^+^ cells from CML patients in chronic phase. (a) Expression of OLFM4 mRNA isoforms in freshly isolated lin^−^CD34^+^ (sCD34+) and sCD34^−^ cells from CML patients and normal controls, as determined by RT-PCR. iCD34^−^ cells were used as a positive control (Pos). L is DNA ladder. (b) qPCR analysis of the expression of OLFM4 mRNA isoforms in sCD34^+^ and sCD34^−^ cells from P1 and P6 CML patients. * p < 0.05 (c) Confocal microscopy showing the expression of OLFM4 in CD34^+^ cells (arrow) in the bone marrow (BM) biopsy specimen from normal donor and CML patient (scale bar = 20 μm). (d) Quantitation of OLFM4 production in sCD34^+^ cultures by ELISA. The lin^−^CD34^+^ cells from patients P1 and P6 were cultured in serum-free medium with 1 ng ml^−1^ SCF, IL3, IL6 and Flt3L with or without 10 ng ml^−1^ G-CSF, 5 μM imatinib, or together for 24 h.

To find out whether the findings obtained using iCD34^+^ cells can be translated to somatic cells, we isolated lin^−^CD34^+^ cells (hereafter referred to as sCD34^+^) form parental bone marrow (patient 1; P1) and bone marrow from several other CML patients in the chronic phase. Using RT-PCR, we detected OLFM4 mRNAs in sCD34^+^ cells isolated from all CML patients. However, in contrast to iCD34^+^ cells generated *de novo* from CML iPSCs, sCD34^+^ cells from CML patients expressed a restricted range of OLFM4 isoforms (Fig. 5a and b). Cytoplasmic OLFM4b isoform was found in sCD34^+^ cells from all CML patients, while none of the patients showed expression of isoform OLFM4e. The expression of cytoplasmic OLFM4c isoform, and secreted OLFM4a and OLFM4d iso-forms varied between patients, but none of the patients expressed the entire range of isoforms. In contrast, sCD34^−^ cells expressed all range of isoforms, although isoform e was not detected in some patients (Fig. 5a and b). None of the OLFM4 isoforms were detectable in normal bone marrow sCD34^+^ cells by PCR (Fig. 5a). To examine whether OLFM4 is expressed in primitive CML cells in situ, we performed immunofluorescent staining of BM biopsies from normal individuals and patients in the chronic phase of CML. As shown in Fig.5c, OLFM4 was detected in CD34^+^ cells from CML patients, but not in normal donors. In contrast, CD34^−^ cells showed OLFM4 protein expression in both normal and CML biopsies. To confirm the production of OLFM4 by the most primitive CML cells, we evaluated OLFM4 secretion by sCD34^+^ (lin^−^CD34^+^) cells isolated from P1 and P6 following the short-term in vitro cultures. Using ELISA, we detected OLFM4 protein in sCD34^+^ cultures from CML patients, while the OLFM4 levels in control BM cultures were not exceeded background level (Fig. 5d). Since previous studies have demonstrated the induction of OLFM4 expression by G-CSF in myeloid progenitors (Chin et al., 2008; Zhang et al., 2002), we tested whether G-CSF enhances OLFM4 production in sCD34^+^ cells from CML patients. We found that G-CSF stimulation indeed enhances OLFM4 production by sCD34^+^ cells and that this effect is potentiated by imatinib (Fig. 5d). To confirm the specificity of G-CSF effect, we cultured sCD34^+^ cells from patient P6 with G-SCF and G-SCF blocking monoclonal antibody. The level of OLFM4 production in presence of antibody was reduced from 205 pg/ml to 47.5 pg/ml.

**Fig. 6:**
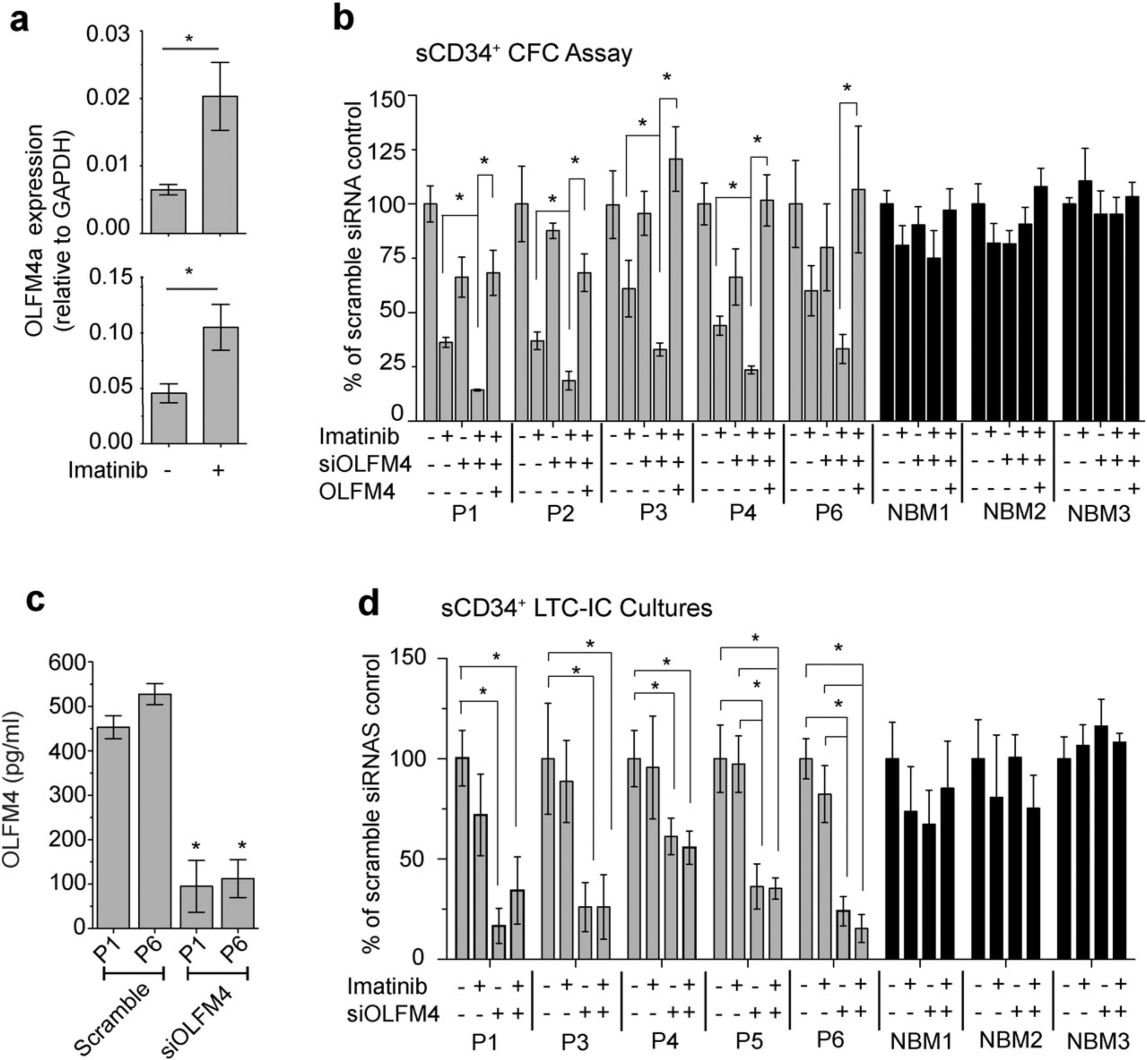
Effects of OLFM4 on somatic lin^−^CD34^+^ CML cells. (a) The effect of imatinib on expression of OLFM4a mRNA isoform in hematopoietic colonies from patients P1 and P6. sCD34^+^ cells were pretreated for 24 h with imatinib and transferred to clonogenic cultures. Hematopoietic colonies were collected seven days after transfection and analyzed by qPCR. Results are mean ± SEM of two experiments performed in triplicates. * p < 0.01 (b) Knockdown of OLFM4 potentiates the inhibitory effect of imatinib on CML sCD34^+^ CFCs (G + GM). After transfection with OLFM4 siRNA (siOLFM4) or scramble (negative control) siRNA, CML or normal bone marrow sCD34^+^ were cultured with or without 5 μM imatinib in presence or absence of OLFM4 protein (300 ng ml^−1^) for 24 h in serum-free medium with low concentrations of growth factors and transferred to clonogenic medium. Results are the mean ± SEM of three to five independent experiments. Significant differences (* p < 0.05) were observed only in CML sCD34^+^ cultures, but not in normal bone marrow sCD34^+^ controls from three patients (NBM1–NBM3). (c) Quantification of OLFM4 protein in the media from 5-week LTC-IC cultures by ELISA. Results are mean ± SEM of three independent experiments. Significant differences (* p < 0.01) were observed in cultures of CML sCD34^+^ transfected with scramble siRNA and siOLFM4. (d) Knockdown of OLFM4 with siRNA dramatically reduced CFC output in LTCIC cultures of CML patients but not of normal bone marrow controls (NBM). Results are mean ± SEM of three experiments. * p < 0.05. NBM = normal bone marrow.

To assess whether OLFM4 provides a survival advantage for CML sCD34^+^ cells, we performed CFC assay after a 24 h treatment with imatinib, siOLFM4, or both. Consistent with prior observations in iCD34^+^ cells, CML cells in clonogenic cultures displayed a significant upregulation of *OLFM4* expression when pretreated with imatinib (Fig. 6a and Supplementary Fig. S5). The OLFM4a mRNA isoform was the most abundant in these cultures (Fig. 6a), while other isoforms were expressed at much lower levels (Supplementary Fig. S5). Knockdown of OLFM4 in CML sCD34^+^ cells using siRNA potentiated the inhibitory effect of imatinib on CFCs, which was reversed by pretreatment of cells with OLFM4 protein (Fig. 6b). In contrast, no significant effect was observed following similar treatments of sCD34^+^ cells from BM of three normal donors (Fig. 6b).

To determine whether OLFM4 provides a survival advantage for cells more primitive than CFCs and whether it can affect the response of these cells to imatinib, we performed an LTC-IC assay using CML sCD34^+^ cells treated with siOLFM4, imatinib or both together. Consistent with previous studies (Bellodi et al., 2009; Corbin et al., 2011), imatinib had little effect on somatic LTC-ICs. In contrast, treatment of cells with siOLFM4 significantly reduced OLFM4 production in sCD34^+^ cultures (Fig. 6c) and the number of colonies in LTC-IC cultures (Fig. 6d and Supplementary Table S3). Addition of imatinib to siOLFM4-treated cultures had no significant impact on LTC-IC numbers (Fig. 6d). Both imatinib and siOLFM4 treatment did not affect substantially the proportion of BCR-ABL positive colonies from LTC-IC cultures (Supplementary Table S4). The observed effect of OLFM4 knockdown on the LTC-ICs was different from the one we observed with less primitive leukemia cells, CFCs. While OLFM4 downregulation alone had minimal effect on CFCs, it significantly augmented the inhibitory effect of imatinib on these progenitors. In contrast, OLFM4 knockdown alone was able to abrogate LTC-ICs and no synergistic action was observed between OLFM4 knockdown and imatinib treatment, thereby suggesting that OLFM4-mediated survival of the most primitive CML cells may not depend on BCR-ABL tyrosine kinase-mediated signaling. Although our studies indicate that knockdown of OLFM4 in sCD34^+^ cells affects primitive CML cell survival in vitro, it remains unclear whether this effect is mediated through cell-intrinsic pathways or through paracrine signaling. Following culture, lin^−^CD34^+^ cells undergo differentiation into lin^+^CD34^−^ cells, that highly express OLFM4. Since siRNAs are transferred to cells following cellular division and the duration of RNA silencing in rapidly dividing cells approaches 1 week (McIntosh et al. 2014), siOLFM4 knockdown most likely reduces OLFM4 production in primitive sCD34^+^ cells as well as in CD34^−^ cells differentiated from sCD34^+^ cells. Thus, OLFM4 secreted by differentiated cells (neoplastic and non-neoplastic) may provide survival cues to primitive CML cells.

**Fig. 7:**
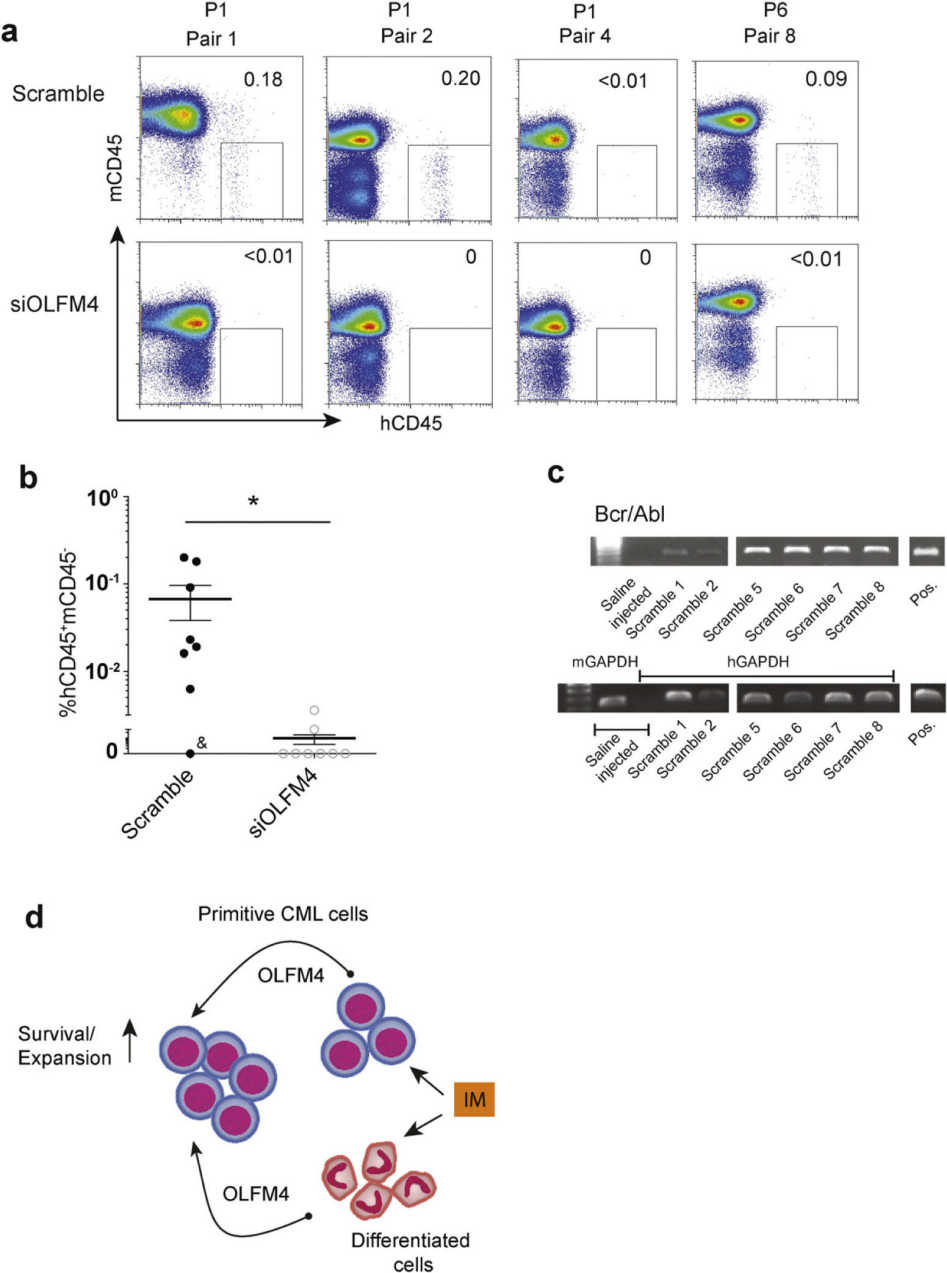
Effect of OLFM4 knockdown with siRNA on in vivo survival of CML sCD34^+^. (a) Representative dot plots show human hCD45 vs. mouse mCD45 labeling in freshly isolated bone marrow samples at 12–14 weeks after transplantation. The samples of sCD34^+^ cells from each CML patient were divided equally, treated with siOLFM4 or scramble siRNA and then injected into retroorbital vein of NBSGW mice. Corresponding mice pairs injected with cells from patient 1 (P1) and 6 (P6) are shown. (b) Frequency of human CD45^+^ cells in bone marrow of NBSGW mice transplanted with scrambled and OLFM4 siRNA. Each point shows individual mouse. & indicates a mice pair injected with 5000 lin^−^CD34^+^ (sCD34^+^) cells. All remaining pairs of mice were injected with 10,000 sCD34^+^ cells. The differences between studied groups are significant (* p = 0.0265). (c) Detection of BCR-ABL expression by RT-PCR in human CD45^+^ cells isolated from bone marrow of six mice injected with scrambled siRNA. Pos = positive control (mRNA isolated from CML iCD34^−^ cells), m = mouse, h = human. (d) The hypothetical model of OLFM4 action on primitive CML cells. OLFM4 produced by CD34^+^ and CD34^−^ cells supports survival and expansion of the most primitive leukemia cells. Since imatinib enhances the production of OLFM4, it can potentially affect the survival of primitive leukemia cells through modulation of OLFM4-mediated signaling.

The ability to engraft in immunocompromised mice is used as important criterion to identify LSC function. However, hematopoietic cells derived from human pluripotent stem cells have markedly impaired engraftment capacity (Slukvin, 2013; Vo and Daley, 2015). Although previous mouse studies have found that forced retroviral expression of BCR-ABL in hematopoietic progenitors generated from pluripotent embryonic stem cells endowed them with engraftment potential (Perlingeiro et al., 2001), we failed to detect meaningful engraftment following transplantation of CML iPSC-derived iCD34^+^ cells (not shown). This could be related to a more “physiological” level of BCR-ABL expression in *de novo* generated human primitive leukemia cells that could be insufficient to enable their engraftment. Therefore, to assess the role of OLFM4 in vivo, we performed transplantation of somatic lin^−^CD34^+^ cells from CML patients in chronic phase using NOD,B6.SCID IL2Rγ^−/−^ KitW41/W41 (NBSGW mice possessing a defective Kit allele; c-kit, W^41^) (McIntosh et al., 2014). Mice models with a defective Kit allele support engraftment of human cells without irradiation, and are superior to irradiated mice for transplantation of human leukemia cells (Cosgun et al., 2014; McIntosh et al., 2014). Using NBSGW mice we were able to detect human CD45^+^ cells in bone marrow following transplantation as low as 10,000 lin^−^CD34^+^ cells from patients in chronic phase of CML. As shown in Fig. 7a and b, most of the NBSGW mice transplanted with scrambled siRNA-transfected CML sCD34^+^ cells demonstrated the presence of human CD45^+^ cells in bone marrow. In contrast, only two out eight mice transplanted with siOLFM4-transfected sCD34^+^ cells showed detectable human CD45^+^ cells at much lower levels compared to corresponding mice in scrambled siRNA group. RT-PCR analysis confirmed that engrafted human CD45^+^ cells expressed BCR-ABL (Fig. 7c). Thus, these studies provide the evidence that OLFM4 is important for the engraftment of primitive leukemia cells in vivo, although it has yet to be determined whether OLFM4 knockdown affects bone marrow homing or in vivo survival of CML lin^−^CD34^+^ cells.

## 3. Discussion

Multiple studies have already demonstrated the validity of the iPSC model for studying pathogenesis of monogenic human diseases and drug screening (reviewed in (Rais et al., 2013)). Although the potential use of iPSC technology in studying neoplasia has been suggested (Ramos-Mejia et al., 2012; Slukvin, 2009; Ye et al., 2009), only a few groups have reported the generation of iPSCs from malignant cells. The first successful reprogramming of cancer cells was achieved by Hochedlinger, who generated mouse pluripotent stem cells by transplanting nuclei from melanoma into oocytes (Hochedlinger et al., 2004). After the discovery of pluripotency factors capable of reprogramming blood cells, iPSC lines have been generated from a patient with a myeloproliferative disorder bearing the JAK2-V617F mutation (Ye et al., 2009), CML cell lines (Carette et al., 2010), primary bone marrow cells of chronic phase CML patients (Bedel et al., 2013; Hu et al., 2011; Kumano et al., 2012), juvenile myelomonocytic leukemia (Gandre-Babbe et al., 2013), and mouse MLL-AF9 leukemia cells (Liu et al., 2013). These studies demonstrated that iPSCs generated from neo-plastic cells carry a disease-specific genetic mutation and can be used to generate blood cells affected by that particular mutation. Here, we advanced the iPSC model to study primitive leukemia cells and proved the utility of this model for discovering drug targets by identifying the novel CML LSC survival factor OLFM4.

The iPSC technology offers several major benefits for studying CML. Current protocols for obtaining primitive CML cells are based on selection of lin^−^CD34^+^ cells from patient bone marrow samples. However, this approach does not reliably isolate LSCs because normal primitive hematopoietic cells usually outnumber BCR-ABL^+^ cells within the lin^−^ CD34^+^ compartment by several folds (Petzer et al., 1996). The capacity of iPSCs to capture CML genetic aberrations, self-renew indefinitely and differentiate into blood cells allows for the production of pure populations of patient-specific BCR-ABL^+^ LSC-like cells in unlimited numbers for functional studies and drug screening. As shown in present studies, the use of pure population of induced lin^−^CD34^+^CD90^+^CD117^+-^CD45^+^CD38^+/−^ BCR-ABL^+^ cells enabled identification of genes affected by imatinib treatment and eventually led to discovery of novel survival factor for primitive leukemia cells OLFM4. iPSCs can be genetically modified to generate CML iPSC lines in which specific gene expression can be conditionally manipulated, thereby facilitating the identification of genes regulating human LSCs’ survival and expansion. iPSCs obtained from the patients before and after acquisition of drug resistance or progression to blast crisis would be a valuable tool for studying the molecular mechanisms of CML progression and drug resistance. Understanding the impact that clonal evolution and diversity has on CML progression requires the selection and expansion of individual leukemia clones; and this represents the major technical challenge. Because iPSCs are of a single cell origin, multiple iPSC lines can be generated from the same patient to capture the diversity of genetic alterations within leukemia cell populations and address the role of non-BCR-ABL-associated mutations in disease development.

Another important finding of this study is the discovery of *OLFM4* as a novel survival factor for primitive CML cells. The human *OLFM4* (also called *GW112* and *hGC-1*) gene encodes a glycoprotein with a multimer structure (Liu et al., 2006). Transcription for this gene produces four spliced variants and one unspliced OLFM4a–OLFM4e mRNA isoforms encoding secreted and cytoplasmic proteins (http://www.ncbi.nlm.nih.gov/IEB/Research/Acembly/av.cgi?db=human&c=Gene&l=OLFM4). OLFM4 plays an important role in a variety of cellular functions including cell adhesion, cell cycle and apoptosis (Tomarev and Nakaya, 2009). In the human intestine, OLFM4 was identified as a robust marker of LGR5^+^ stem cells and a subset of cancer cells (van der Flier et al., 2009). OLFM4 is involved in cell growth and apoptosis in human malignancies (Liu et al., 2010; Oh et al., 2011; Park et al., 2012; Zhang et al., 2004) and is considered to be an inducible resistance factor to apoptotic stimuli (Koshida et al., 2007). OLFM4 interacts with GRIM-19 (Zhang et al., 2004), which is a component of the respiratory complex I of mitochondria with an anti-apoptotic role in prostate cancer cells (Huang et al., 2004). However, the effects of OLFM4 on apoptosis appear to be a cell-type dependent. In contrast to prostate cancer cells, overexpression of OLFM4 in HL-60 leukemia cells induces their differentiation and apoptosis (Liu et al., 2010). Although OLFM4 was initially identified in myeloblasts and later in neutrophils (Clemmensen et al., 2012; Zhang et al., 2002), OLFM4's biological function in normal and neoplastic hematopoiesis remains largely unknown. It has been shown that OLFM4 expression is upregulated in a subset of patients with acute myeloid leukemia and primary myelo-fibrosis (Hasselbalch et al., 2014; Liu et al., 2010). Here, we demonstrated the expression of OLFM4 protein in lin^−^CD34^+^ and CD34^−^ bone marrow cells from CML patients in chronic phase and revealed the distinct pattern of OLFM4 isoform expression in these cells at the mRNA level. CML lin^−^CD34^+^ cells expressed predominantly cytoplasmic OLFM4 mRNAs. In contrast, CML CD34^−^ differentiated cells displayed the broad spectrum of cytoplasmic and secreted OLFM4a–OLFM4d isoforms. Using OLFM4 knockdown studies, we discovered the critical role OLFM4 in the survival and drug resistance of primitive CML cells and demonstrated differences in OLFM4-mediated regulation of cell survival in CFCs and more primitive LTC-ICs (Fig. 7d). While OLFM4 had a minimal role in CML CFC maintenance in normal conditions, it was required for protection of CML CFCs from imatinib-mediated inhibition. In contrast, maintenance of CML LTC-ICs was dependent on OLFM4 and independent of BCRABL kinase activity. Consistent with in vitro LTC-IC studies, OLFM4 knockdown also abrogated the engraftment of BCR-ABL^+^ cells in NBSGW mice, thereby providing another critical evidence for the essential role of OLFM4 in homeostasis of CML cells in vivo. The mechanisms responsible for the distinct role of OLFM4 in the survival of leukemia cells at different stages of maturation remain to be investigated. It is possible that cell-context specific effects may be related to differences in the expression and/or regulation of the OLFM4 isoforms in a stage-specific fashion. It is also important to investigate whether OLFM4 mediates cell survival in a self-autonomous or non-autonomous manner. Given that lin^−^CD34^+^ cells acquire a CD34^−^ differentiated phenotype which is associated with a high level of OLFM4 expression in culture, it is possible that secretion of OLFM4 by differentiated cells generates a milieu supportive for the growth of undifferentiated CML cells in vitro. Similarly, we observed that differentiated CD34^−^ cells next to CD34^+^ cells in bone marrow biopsy samples of CML patients express high levels of OLFM4. In addition, OLFM4 is produced by normal non-leukemic CD34^−^ cells. Thereby, OLFM4 secreted by differentiated leukemic or nonleukemic cells may confer a selective growth advantage to the more primitive CML cells and LSCs in vivo.

Overall, our studies indicate that OLFM4-mediated signaling supports the survival of primitive leukemia cells. Thus, analysis of OLFM4 and its isoforms in peripheral blood could be exploited as predictive markers for treatment regimens, while targeting OLFM4 or OLFM4-mediated signaling could be explored as an option to eradicate primitive CML cells. Potentially, OLFM4 could be targeted using anti-OLFM4 antibodies. An antibody approach has already been described to modulate the activity of another protein within the OLFM family, OLFM3. As shown by Miljkovic-Licina (Miljkovic-Licina et al., 2012), administration of anti-OLFM3 antibodies efficiently blocked proangiogenic signaling mediated by this protein in vivo. Finally, our studies provide a strong background and novel model for the further exploration of the mechanisms of OLFM4 action in primitive CML cells.

## 4. Material and methods

### 4.1. iPSCs maintenance and hematopoietic differentiation

In this study, we used previously described transgene-free BM9, CML15 and CML17 iPSCs produced by reprogramming of normal bone marrow mononuclear cells and bone marrow mononuclear cells from patient with newly diagnosed CML in the chronic phase (Hu et al., 2011). BM1K iPSC was generated from the normal control using the same approach (Hu et al., 2011). Undifferentiated iPSCs were maintained in cocultures with mouse embryonic fibroblasts (MEFs). Hematopoietic differentiation was induced by transferring the iPSCs to overgrown OP9 feeders as we have previously described in detail (Choi et al., 2009a; Vodyanik et al., 2005). VEGF (100 ng ml^−1^) was added to some cultures at initiation of differentiation to enhance blood production. CD43^+^ cells were collected on day 9 of differentiation using MACS and cultured in α-MEM supplemented with 10% FBS, 50 μg ml^−1^ ascorbic acid, 100 μM monothioglycerol (complete serum supplemented medium (CSSM)) and 200 ng ml^−1^ GM-CSF to selectively expand myeloid progenitors (Choi et al., 2009a). After two days of expansion with GM-CSF CD43^+^ cells enriched in myeloid progenitors were cultured for an additional four days in the same media supplemented with 10 ng ml^−1^ IL-3, 100 ng ml^−1^ IL-6, 100 ng ml^−1^ Flt3L, 100 ng ml^−1^ SCF, and 200 ng ml^−1^ GM-CSF (all from Peprotech).

### 4.2. Isolation of lin^−^CD34^+^ cells from iPSC cultures

CD43^+^ hematopoietic cells were collected from differentiated iPSC cultures using MACS and labeled with CD235a/CD41a FITC, CD45 APC and CD38 PE (BD Pharmingen). Lin-CD45+CD38+ and lin-CD45+CD38-subpopulations were obtained by fluorescence-activated cell sorting using FACSAria (BD) (Vodyanik et al., 2006).

### 4.3. Collection of bone marrow specimens from CML patients in the chronic phase, and controls, and purification of lin-CD34^+^ cells

Bone marrow mononuclear cells from CML patients in the chronic phase were purchased commercially (AllCells or Applied StemCells), or obtained from the patients at the University of Wisconsin Hospital and Clinics (Madison, WI) with approval from the University of Wisconsin Institutional Review Board. Donors had previously signed an Institutional Review Board-approved consent. Patients P1, P2, P5–P7 were newly diagnosed with CML. All studied (P1–P7) patients were sensitive to imatinib. Bone marrow cells from healthy donors were obtained from Cincinnati Children's Hospital Medical Center (CCHMC). Mononuclear cells were labeled with the lineage-specific markers CD2, CD3, CD14, CD15, CD16, CD19, CD20, CD24, CD41a, CD56, CD66b, and Glycophorin A (FITC-conjugated antibodies), CD34 APC (BD Pharmingen) and DAPI to exclude dead cells. Live lin^−^CD34^+^ cells were isolated using FACSAria (BD). In some experiments, mononuclear cells were thawed and cultured overnight followed by labeling and isolation of lin^−^CD34^+^ cells as described above.

### 4.4. Hematopoietic colony-forming assay

Hematopoietic clonogenic assays were performed using serum-containing StemMACS semisolid clonogenic medium (Miltenyi Biotec, CA). Cells were incubated with either DMSO or 5 μM imatinib in serum-free medium (SFM) composed of IMDM, 10% BIT (Stem Cell Technologies), 2-mercaptoethanol, and EXCYTE (Millipore) and supplemented with a low concentration of growth factors (1 ng ml^−1^ of each SCF, IL3, IL6, Flt3L and GM-CSF). After 24 h of cultures, cell collected and plated in clonogenic medium without imatinib.

### 4.5. Rhodamine 123 exclusion aldehyde dehydrogenase activity assays

Rho exclusion assay was performed as previously described (Vodyanik et al., 2005). Aldehyde dehydrogenase (ALDH) staining was performed with the Aldefluor kit (Stem Cell Technologies) per manufacturer instructions.

### 4.6. Apoptosis assay

CML iCD34^+^ cells were cultured in CSSM containing 10 ng ml^−1^ IL3, 100 ng ml^−1^ IL6, 200 ng ml^−1^ GM-CSF, 100 ng ml^−1^ SCF, and 100 ng ml^−1^ Flt3L with or without 5 μM imatinib for 24 h before analysis of apoptosis. When indicated, cells were transfected with either OLFM4 or scrambled (control) siRNA. Cells were stained with Annexin-V–PE and 7-AAD using the Annexin V: PE Apoptosis Detection Kit (BD Bioscience) according to the manufacturer's protocol and analyzed by flow cytometry. In some experiments green Caspase 3/7 detection reagent (Life Technologies) was used in combination with Annexin-V-PE staining to detect apoptotic cells.

### 4.7. Western blotting

Cells were cultured in serum-free medium without growth factor in the presence or the absence of 5 μM imatinib for 4 h prior to harvesting. Lysates were prepared in RIPA buffer containing 1% Nonidet P-40 and 1% sodium deoxycholate supplemented with 1 mM phenylmethylsulfonyl fluoride, protease inhibitors cocktail and 1 mM sodium vanadate. Proteins were separated by SDS-PAGE, transferred to nitrocellulose membrane and immunoblotted for phospho-CRKL (py207), phospho-Abl (py245) and phospho-BCR-ABL (pY245) antibodies (Cell Signaling Technology). Signals were detected with HRP-conjugated secondary antibody using the ECL kit (Amersham). CML cell line K562 was included as a positive control. p-CRKL expression levels were determined by densitometry using ImageJ software (NIH).

### 4.8. Cell proliferation assay

Total lin^−^CD34^+^, lin^−^CD34^+^CD38^−^ or lin^−^CD34^+^CD38^+^ were plated in triplicate in 96-well plate at 10^3^ cells/well. Cells were then cultured in SFM with or without 5 μM imatinib. When specified, the 300 ng ml^−1^ of OLFM4 (Acro Biosystem) or the following growth factors were added: 10 ng ml^−1^ IL3, 100 ng ml^−1^ SCF, 100 ng ml^−1^ Flt3L, 100 ng ml^−1^ IL6, and 200 ng ml^−1^ GM-CSF. Viable cell yields were determined by counting trypan blue excluding viable cells using a hemocytometer.

### 4.9. Long-term culture initiating cell assay (LTC-IC)

Sorted iPSC-derived lin^−^CD34^+^CD45^+^ cells were plated in a six-well plate at 10^4^ cells/well containing 5–7 day-old cultures of murine 1 × 10^5^ M2-10B4 and OP9 stromal cells (1:1 ratio mix) in a LTC-IC medium consisting of SFM supplemented with 10 μM hydrocortisone, 50 ng ml^−1^ SCF, 5 ng ml^−1^ IL3, and 50 ng ml^−1^ IL6. Cultures were maintained at 37 °C in a humidified atmosphere with 5% CO_2_, and fed at weekly intervals. After five weeks, cells were harvested and analyzed for CFC potential as described above. LTC-IC assay for somatic lin^−^ CD34^+^CD45^+^ cells was performed using M2-10B4 cells exactly as described in the Stem Cell Technologies protocol (http://www.stemcell.com/en/Technical-Resources/db5a9/28412_ltc_ic-H.aspx). When indicated, cells were pretreated for one week with 5 μM imatinib or DMSO (control), in SFM supplemented with low concentration of growth factors (1 ng ml^−1^ of each SCF, IL3, IL6, Flt3L and GM-CSF), and transferred to LTC-IC cultures for an additional five weeks of culture. In some experiments, cells were transfected with either OLFM4 or negative control siRNA and then pretreated with imatinib or DMSO as described above.

### 4.10. IC_50_ assay

The lin^−^CD34^+^ and lin^+^CD34^−^ cells were plated at 10^3^ cells/well in a 96-well plate in CSSM containing 10 ng ml^−1^ IL3, 100 ng/ml IL6, 200 ng ml^−1^ GM-CSF, 100 ng ml^−1^ SCF, and 100 ng ml^−1^ Flt3L with 0–100 μM imatinib. After 24 h of culture, a viable cell count was performed using trypan blue. The IC_50_ was determined as the drug concentration where cell death was 50% of maximum in the upper plateau (Sebaugh, 2011). Data from three assays performed in triplicate were used for statistical analysis and graph plots for IC_50_ determinations. Relative IC_50_ were determined by fitting an exponential dose–response curve to the cell proliferation data by using GraphPad Prism software (GraphPad, San Diego, CA).

### 4.11. CFSE tracking of cell division

Differentiated cells were labeled with 1 μM carboxy-fluorescein diacetate succinimidyl diester (CFSE; Molecular Probes, Eugene, OR) as previously described (Copland et al., 2006; Holtz et al., 2002). These cells were then incubated overnight in CSSM medium supplemented with growth factors to allow excess unbound dye to leak out of the cells. Cells cultured in the presence of 10 μg/ml mitomycin C (Sigma Aldrich) were used to establish the CFSE_max_ (undivided cell population). The next day, CFSE^bright^ cells were sorted by FACArias to exclude non-labeled and CFSE^dim^ populations. These cells were then cultured for four days in CSSM supplemented with growth factors with or without 5 μM imatinib. At the end of the culture period, cells were stained with CD34-APC and 7AAD for flow cytometry analysis. The percentage of cells in each generation was determined using FlowJo software (Tree Star, Ashland, OR), with the position of the parent generation set on the basis of the fluorescence profile of undivided cells.

### 4.12. Adhesion assay

The lin^−^CD34^+^CD45^+^ cells were incubated in SFM supplemented with 1 ng/ml each of SCF, IL-3, IL-6, Flt3L and GM-CSF, with and without 5 μM imatinib for 24 h. Cells were then washed and resuspended in SFM and plated onto either fibronectin- or BSA-coated wells of 96-well plates at 10^3^ cells/well. After 2 h, nonadherent and adherent fractions were separated as described previously (Bhatia et al., 2001; Holtz et al., 2002). Both fractions were plated in serum-containing StemMACS HSC-CFU medium (Miltenyi Biotec, CA), and the percentage of CFCs in adherent fraction was calculated.

### 4.13. Immunofluorescence microscopy

Heat-induced antigen-retrieved paraffin sections of bone marrow biopsy from normal subjects and CML patients (N = 2 each) were stained with mouse anti-human CD34 (Ventana), and rabbit anti-human OLFM4 (Abcam), or matched IgG isotype primary antibodies. After staining with secondary antibodies, anti-mouse IgG Alexa Fluor 555 and anti-rabbit IgG Alexa Fluor 488 (Life Technology), and DAPI, pictures were acquired by Nikon Eclipse Ti-E configured with an A1R confocal system (Nikon Instruments Inc. Melville, NY) and Nikon Elements (NIS — element C) imaging software (Nikon Instruments Inc. Melville, NY).

### 4.14. Detection of OLFM4 by ELISA

The lin^−^CD34^+^ cells from patients P1 and P6 (2000 cells per well) were cultured in 120 μl serum-free medium with 1 ng ml^−1^ SCF, IL3, IL6 and Flt3L with or without 10 ng ml^−1^ G-CSF, 5 μM imatinib, or together for 24 h. For G-CSF neutralization, cells treated with cultures treated with 0.03 μg ml^−1^ mouse human G-CSF neutralizing antibody (clone #3316 R&D Systems). OLFM4 protein in cultures was quantified using a sandwich ELISA kit (USCN Life Science) according to manufacturer instruction.

### 4.15. siRNA transfection

OLFM4 siRNA composed of four *OLFM4*-specific siRNAs (siOLFM4) or control scrambled siRNA (AllStars Neg. siRNA AF 488) were obtained from Qiagen. iPSCs-derived lin^−^CD34^+^CD45^+^ and somatic CML bone marrow lin^−^CD34^+^ cells were transfected with a total 100 nM of either OLFM4 or scrambled siRNA using the HiPerfect transfection reagent according to the manufacturer's protocol (all from Qiagen). The transfection efficiency was 50–60% as evaluated by using control ALLStars Neg. siRNA AF488. As determined by qPCR performed 24 h after transfection, the silencing efficiency was 60–85% (Supplementary Fig. S4b).

### 4.16. Gene expression analysis by real-time PCR (qPCR)

RNA was isolated from the cell subpopulations using the PureLink RNA mini kit (Life Technologies). cDNA synthesis was carried out using Advantage RT-for-PCR kit (Clontech). Quantitative real-time PCR analysis was performed using the PlatinumSYBR Green qPCR SuperMix-UDG kit (Life Technologies) and primers listed in Supplementary Table S5. The reactions were run on a Mastercyclerep realplex thermal cycler (Eppendorf) and expression levels were calculated by minimal cycle threshold values (Ct) normalized to the reference expression of GAPDH in each sample (Pfaffl, 2001). When specified, K562 was used as a reference. All qPCR products were analyzed on 1.2% agarose gels to confirm the specificity of detection.

### 4.17. RNA-Seq analysis

Day 11 iCD34^+^ cells were isolated and cultured CSSM supplemented with 200 ng ml^−1^ of GM-CSF with DMSO or 5 μm of imatinib. After 16 h, total RNA from cells was isolated with PureLink RNA mini kit (Life Technologies) and treated with DNaseI TURBO DNase™ kit (Ambion). Total RNA was quantitated using the Life Technologies Qubit fluorometer (Q32857) and the Agilent Bioanalyzer 2100. Samples were then prepared for sequencing using the Illumina TruSeq RNA Sample Preparation Kit v2 (RS-122-2001), according to the manufacturer's protocol. Final sample libraries were quantitated with the Life Technologies Qubit fluorometer and sequenced on the Illumina HiSeq 2500 (SY-401-1003-PRE). Base-calling and demultiplexing were done with the Illumina Genome Analyzer Casava Software, version 1.8.2. Following quality assessment and filtering for adapter molecules and other sequencing artifacts, the remaining sequencing reads were aligned to 19,084 RefSeq genes extracted from the Illumina iGenomes annotation, selecting only “NM_” designated genes. Bowtie v 0.12.9 was used, allowing two mismatches in a 28 bp seed, and excluding reads with more than 200 alignments (Langmead et al., 2009). RSEM v 1.2.3 was used to estimate isoform or gene relative expression levels in units of “transcripts per million” (tpm) (Li and Dewey, 2011; Li et al., 2010). To determine differentially expressed genes, RNAseq output data were analyzed using EBseq(v.1.1.6) http://www.biostat.wisc.edu/~kendzior/EBSEQ/ (Leng et al., 2013). Genes with a posterior probability equal to 1.000 were considered differentially expressed. Genes with tpm < 10 across all studied samples were excluded from analysis. Remaining genes demonstrating significant differences in expression between the studied groups were assigned to biological process categories using the DAVID bioinformatics program (Huang da et al., 2008). To visualize the gene-expression levels, a heat-map was composed using MultiExperiment Viewer v4.2 (http://www.tm4.org). PCA was performed with TPM (transcripts per million) normalized data using “stats” package for R programming language, with “scale” option disabled. The data was preliminary restricted to genes that were called differentially expressed between CML-control and CML-Imatinib with Benjamini-Hochberg false discovery rate below 0.01.

### 4.18. Animal transplantation

Engraftment of sCD34^+^ cells was evaluated using NBSGW mice possessing a defective Kit allele; (c-kit, W^41^) (McIntosh et al., 2014). Bone marrow lin^−^CD34^+^ cells from the CML patients in chronic phase were equally divided and transduced with scramble siRNA or siOLFM4. 5000 or 10,000 lin^−^CD34^+^ cells were injected into the retroorbital vein of NBSGW mice. Animals were analyzed at 12–16 weeks after transplantation by flow cytometry and presence of BCR-ABL transcripts was confirmed by RT-PCR. To evaluate BCR-ABL expression in engrafted cells, bone marrow samples from transplanted animals were cultured in SFM with human SCF, IL3, IL6 and Flt3L (each at 100 ng ml^−1^) to expand human cells. After 6–7 days of culture, human CD45^+^ cells were isolated by FACS and analyzed by for BCR-ABL expression by RT-PCR.

### 4.19. Statistical analysis

Data obtained from multiple experiments were reported as the mean ± SEM. Significance levels were determined by one-tailed Student-*t* test analysis.

## Supplementary Material

Figures

Table 1

Table 2
