# Supplementary material for: Discovery of survival factor for primitive chronic myeloid leukemia cells using induced pluripotent stem cells
Source: Stem Cell Res. Author manuscript; Available in PMC 2016 Aug 29. (PMC5003778; doi:10.1016/j.scr.2015.10.015)
Supplement: Figures [file NIHMS810460-supplement-Figures.pdf]

Supplementary Figures

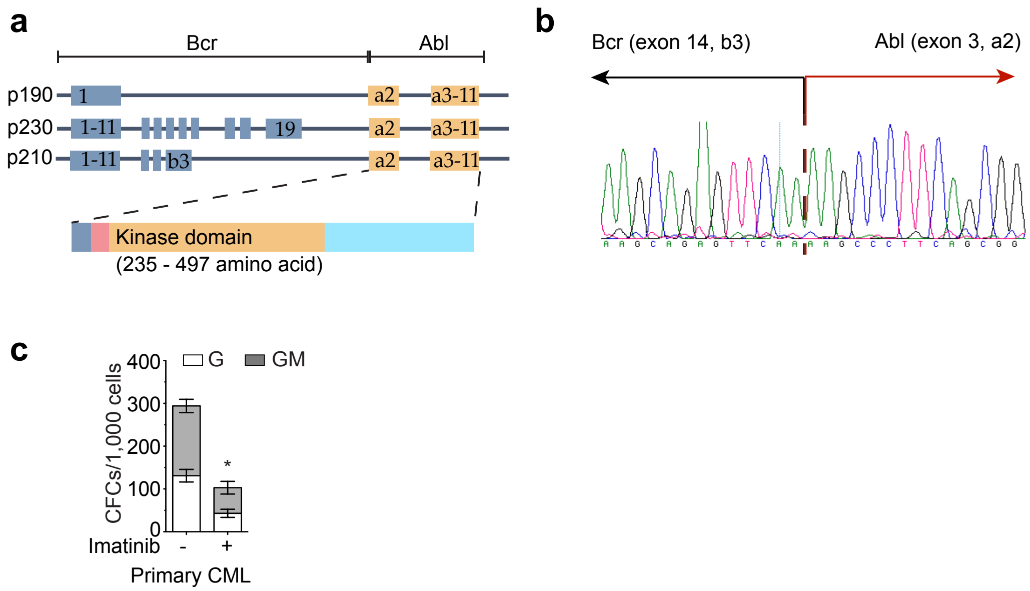

Supplementary Figure S1. Characterization of BCR-ABL fusion in iPSCs and imatinib sensitivity of paternal CML bone marrow cells. (a) Schematic diagram showing three different isoforms of the BCR-ABL protein. **(b)** CML iPSCs expressed the typical p210 isoform without mutation in the kinase domain (235 – 497 amino acid). Flow chromatogram depicts the site of translocation junction. **(c)** The effect of imatinib pretreatment on myeloid CFC potential of the parental $lin^{-}CD34^{+}$ BM cells from CML patient used to generate iPSCs. Somatic $lin^{-}CD34^{+}$ cells were incubated 24 hours in serum-free medium with low concentration of growth factors with or without 5 μM imatinib and then transferred to CFC medium. Results are mean $\pm$ SEM of three experiments. * $p < 0.05$.

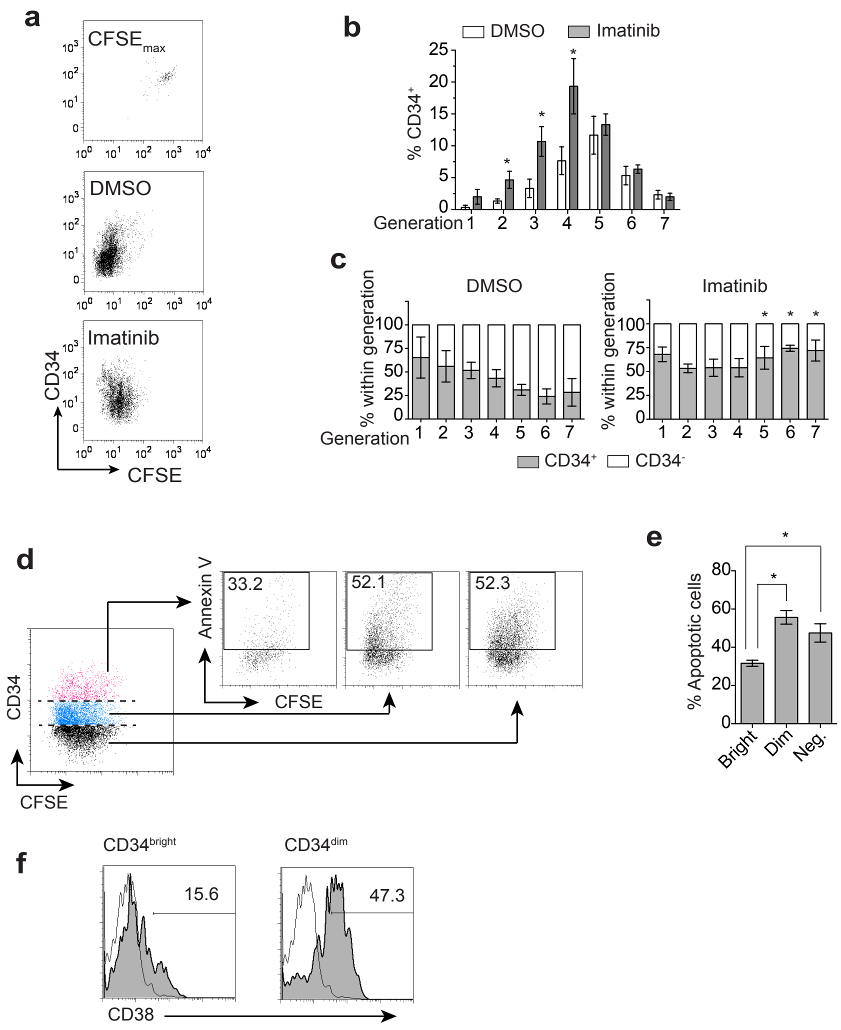

Supplementary Figure S2. CFSE tracking of CML and BM iCD34$^+$ cells after treatment with imatinib. (a) iCD34$^+$ cells were cultured with or without 5 µM imatinib for four days. Mitomycin C-treated cells were used to set the CFSE$_{max}$. Dot plot shows CD34 vs CFSE. **(b)** Histogram shows the percentage of viable CD34$^+$ cells from each generation in the total culture as determined by CFSE tracking. iCD34$^+$ cells were labeled with CFSE and cultured with growth factors with or without 5 µM imatinib for four days. At the end of the culture period, cells were stained with CD34-APC and 7AAD for flow cytometry analysis. The percentage of cells in each generation was determined using FlowJo software**. (c)** The relative proportion of CD34$^+$ and CD34$^-$ cells within each generation in control and imatinib treated cultures. Bars show mean ± SEM of three experiments. * indicates significant difference ($p<0.05$) between imatinib treated and corresponding DMSO cultures. **(d)** Flow cytometric analysis of apoptosis (7AAD$^-$Annexin V$^+$) and CD34 expression at the end of 4 days expansion with 5 µM imatinib. **(e)** Chart shows percentage of apoptotic cells within CD34^{bright}, CD34^{dim}, and CD34$^-$ (negative) populations gated from left dot plot. Results are mean ± SEM of three experiments. * $p<0.05$. **(f)** CD38 expression by CD34^{bright} and CD34^{dim} cells.

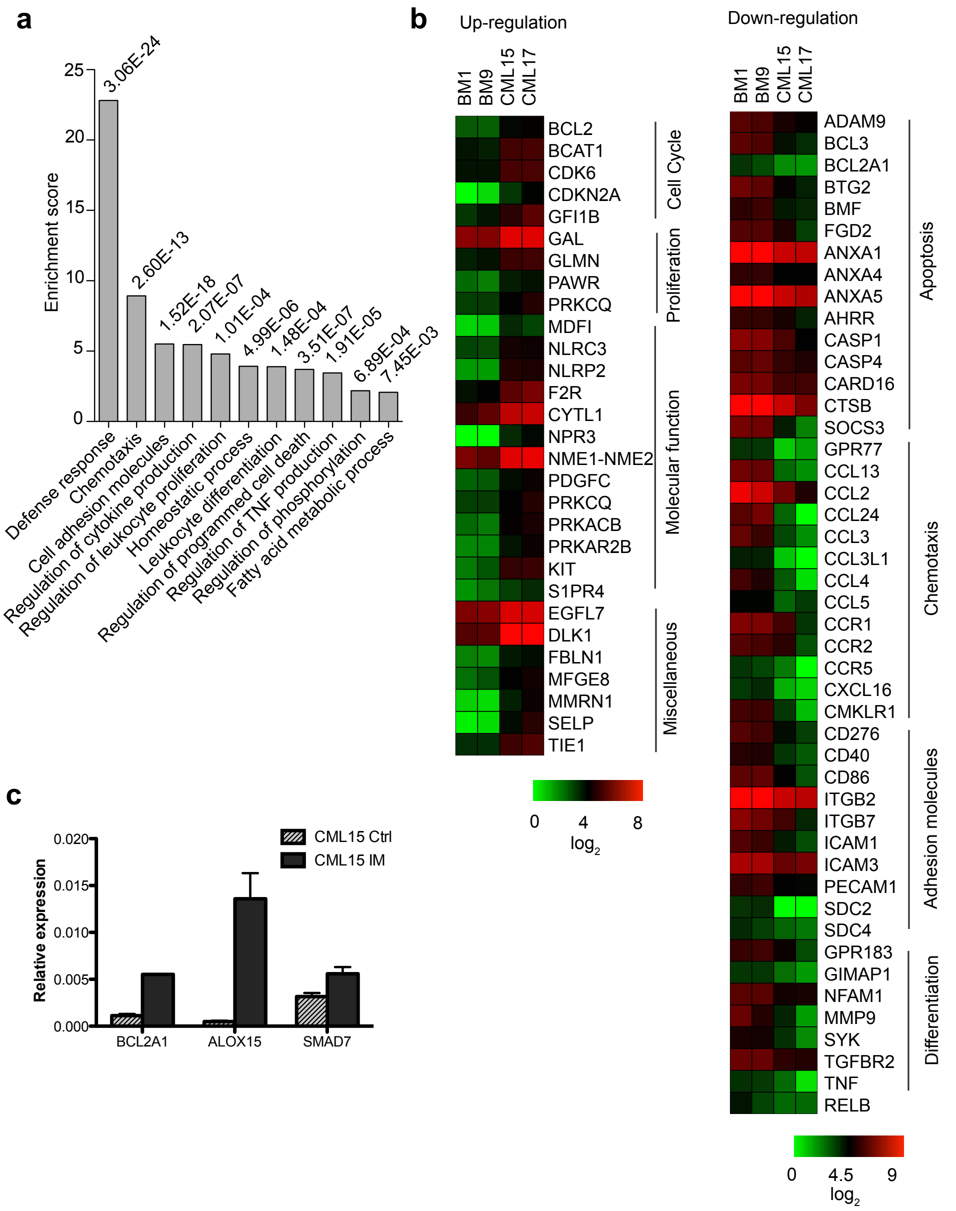

Supplementary Figure S3. Molecular profile of BM and CML iCD34$^+$. (a) The 922 differentially expressed genes between CML and BM iCD34$^+$ cells showing ≥ 2 fold change were classified into biological processes as defined by the Gene Ontology (GO) Term using DAVID program. Representative clusters are shown in a histogram along with p-value. **(b)** Heat maps show selected up-regulated and down-regulated genes in CML iCD34$^+$ within identified GO categories. The gene expression levels are estimated in tpm. **(c)** qPCR analysis of BCL2A1, ALOX15 and SMAD7 expression in CML15 iCD34$^+$ cells.

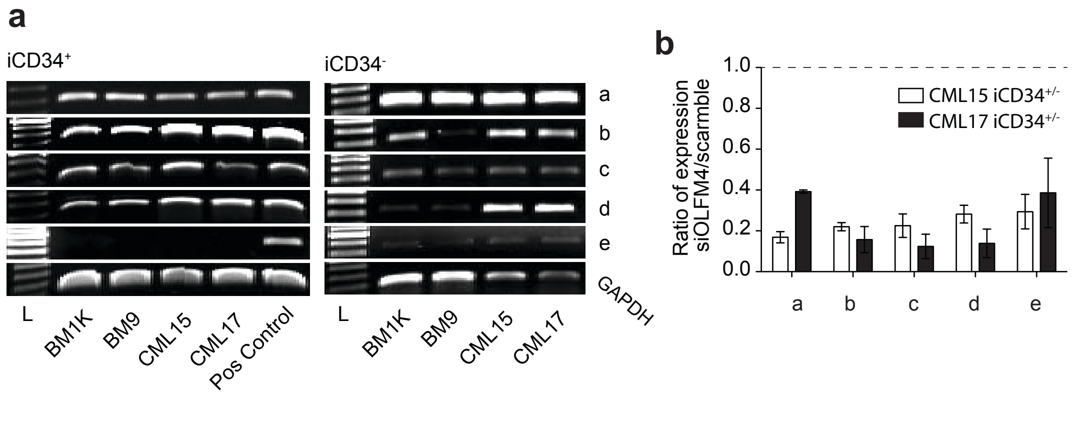

Supplementary Figure S4. OLFM4 expression in iCD34$^+$ and iCD34$^-$ cells generated from CML and normal BM iPSCs and OLFM4 knockdown efficiency with siRNA. (a) Expression of OLFM4 mRNA isoforms in control (BM) and CML iCD34$^+$ and iCD34$^-$ cells as determined by RT-PCR. L is DNA ladder. Positive control in left panel shows iCD34$^-$ cells. **(b)** qPCR analysis of the knockdown efficiency of OLFM4 mRNA isoforms in CML iPSC-derived hematopoietic cells (CML iCD34$^{+/-}$) using OLFM4 siRNA (siOLFM4). Results are mean ± SEM of 3 independent experiments in duplicate. The expression level was calculated relative to negative control (scramble siRNA) 24 hours after transduction.

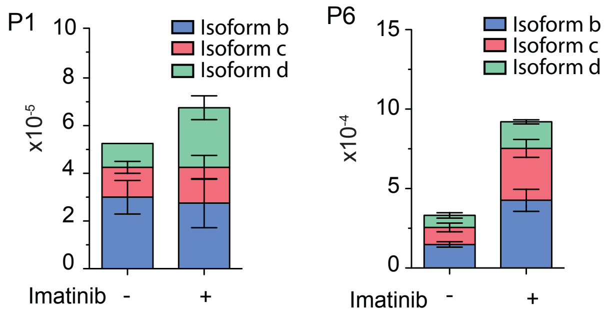

Supplementary Figure S5. qPCR analysis of the expression of OLFM4b-OLFM4d mRNA isoforms in hematopoietic colonies from CML P1 and P6 patients. sCD34^{+} cells were pretreated with imatinib or DMSO (control) for 24 hour and placed into clonogenic medium. After 7 days of clonogenic culture colonies were collected and analyzed by RT-PCR.

Supplementary Table S3. Absolute numbers of CFCs from LTC-IC cultures of sCD34$^+$ cells treated with imatinib (IM) and siOLFM4.

	Total number of CFCs per 1000 initial lin$^-$CD34$^+$ cells (mean±SEM)			
	Scramble	Scramble+IM	siOLFM4	siOLFM4+IM
CML P1	176±35	136±38	31±17	68±28
CML P3	219±60	193±45	57±27	57±35
CML P4	424±59	405±110	261±39	237±35
CML P5	242±40	236±34	88±27	72±27
CML P6	267±27	220±39	64±20	41±18
NBM1	228±42	168±51	153±39	195±53
NBM2	231±45	185±72	232±26	174±38
NBM3	308±34	328±32	358±41	333±14

Supplementary Table S4. Percentage of BCR-ABL positive colonies from LTC-IC cultures of sCD34$^+$ cells treated with imatinib (IM) and siOLFM4.

	Baseline	Scramble+IM	siOLFM4+IM
CML P1	70	90	80
CML P6	90	80	60

Supplementary Table S5. Primers used in the study

Gene	Reference Sequence	Forward	Reverse	Product Size
hGAPDH	NM_001256799.2	GTGGACCTGACCTGCCGTCT	GGAGGAGTGGGTGTCGCTGT	153
mGAPDH	NM_001289726.1	AGACCACAGTCCATGCCA	TTGCCCACAGCCTTGGCA	137
OLFM4a	NCBI AceView OLFM4; aAug10	CCGTGGACAGAGTGGAAC	TCTACCTTGATCAGCTCGAA	202
OLFM4b	NCBI AceView OLFM4; bAug10	ACTTGAGGGTGGAGGGTGA	ATTTTGCTTCAGCATCATCATTTGC	217
OLFM4c	NCBI AceView OLFM4; cAug10-unspliced	ATAGTTCTTGCTGGTTTCATTATTC	TAATATCCTAAGCACCAAAGGAAT	214
OLFM4d	NCBI AceView OLFM4; dAug10	ACCTCCTGGGGCAGTTCA	CACATCCCCCAAATCCCCT	211
OLFM4e	NCBI AceView OLFM4; eAug10	ATGTATTGGACTCCACTTACTT	CATGATGTCAATTCGGACAGT	254
OLFM4a/b	NCBI AceView OLFM4; aAug10 and bAug10	CCAGCTGGAGGTGGAGATAAG	TCAGAGCCACGATTTCTCGG	103
BCR/ABL	Bcr/Abl NM_004327.3 NM_005157.5	ACGTTCCTGATCTCCTCTGACTATG	TCAGACCCTGAGGCTCAAAG	251

NCBI AceView
http://www.ncbi.nlm.nih.gov/IEB/Research/Acembly/av.cgi?db=human&c=Gene&l=OLFM4
